# Small Relative Age Effect Appears in Professional Female Italian Team Sports

**DOI:** 10.3390/ijerph19010385

**Published:** 2021-12-30

**Authors:** Paolo Riccardo Brustio, Gennaro Boccia, Paolo De Pasquale, Corrado Lupo, Alexandru Nicolae Ungureanu

**Affiliations:** 1 Department of Neuroscience, Biomedicine and Movement, University of Verona, 37131 Verona, Italy; paoloriccardo.brustio@univr.it; 2 Neuro Muscular Function Research Group, School of Exercise & Sport Sciences, University of Turin, 10143 Turin, Italy; gennaro.boccia@unito.it (G.B.); paolo.depasquale@unito.it (P.D.P.); alexandru.ungureanu@unito.it (A.N.U.); 3 Department of Clinical and Biological Sciences, University of Turin, 10143 Turin, Italy; 4 Department of Medical Science, University of Turin, 10143 Turin, Italy

**Keywords:** gender difference, RAE, talent development, team sports

## Abstract

The relative age effect (RAE) concerns those (dis)advantages and outcomes resulting from an interaction between the dates of selection and birthdates. Although this phenomenon is well known in a male context, limited data are available in female sports. Thus, the aim of this study was to quantify the prevalence and magnitude of the RAE in a female Italian context at the professional level in basketball, soccer, and volleyball. A total of 1535 birthdates of elite senior players were analyzed overall and separately between early and late career stages. Chi-square goodness-of-fit tests were applied to investigate the RAE in each sport. An asymmetry in birthdates was observed in all sports (Crammer’s V ranged = 0.10–0.12). Players born close to the beginning of the year were 1.62 and 1.61 times more likely to reach first and second Italian divisions of soccer and volleyball, respectively, than those born in the last part of the year. A small over-representation of female athletes born close to the beginning of the year is evident at the senior professional level in all Italian investigated team sports. In soccer, this trend was more evident in the first stage of a senior career.

## 1. Introduction

Sport organizations usually group children and adolescents into homogeneous chronological age cohorts (generally of one or two years) to create age bands and competitive tiers. The purpose of this choice is to guarantee fair learning opportunities and competitive experiences by limiting massive intragroup physical and cognitive differences, especially in invasion team sports. Nevertheless, an interval of one year within the same age cohort may create developmental differences, as well as participation and attainment among peers with an advantage for the athletes born in the first months of the selection year [1,2]. The relative age effect (RAE) concerns those (dis)advantages and outcomes resulting from an interaction between the dates of selection and birthdates [3]. As a consequence of RAE, an over-representation of chronologically older athletes within the same age-grouping cohort was observed.

One of the most supported explanations about RAE in sport is the maturation-selection hypothesis [1], which assumes the enhanced physical and anthropometric characteristics (i.e., stature, mass, speed, muscular strength, and aerobic power) of chronological older children/youth. Moreover, the influence of social agents, like parents and coaches, should also be considered. These agents may focus their attention on the relatively older athletes due to misguided interpretations of talents based on size/age and may create and exacerbate RAE [4,5].

The differences in terms of biological age across young athletes [6] may accentuate physical, cognitive, and psychological differences [1,7] and influence the talent selection process, as well the development of athletes’ abilities/skills. Therefore, the younger athletes have less opportunities to reach the highest levels in elite sports and consequently, drop out [8,9]. In other words, athletes born close to the selection date achieve advantages in developmental ages in term of sport success and process of talent identification [2,10]. This is particularly evident also considering the first stage of career progression, where being relatively older at the beginning of the career may possibly increase the chance to outperform the peers and reach success during junior career [11,12,13], especially in high physical demanding sports. In this regard, the RAE at the senior level in different team sports, including soccer, rugby, volley, and basketball, is greater in players at the beginning of their career (i.e., athletes of about 21 years old) than in the later phase [14].

In a male context, the magnitude of RAE is well documented, especially in sports where physical factors are fundamental for success [1] affecting a wide range of individual [6,11,12,15] and team sports from childhood to adulthood [14,16,17,18,19,20]. However, RAE may be influenced by sex differences. RAEs are more variable in female sports in comparison with male sports [3] and its presence in female sport context is still widely discussed [8]. The RAE has a smaller magnitude on female sports whilst it is modulated by the same factors as for males (i.e., participant’s age, competition level, sport type, and sport context) [1]. In fact, similarly to males, RAE in female competitions increases in the youngest age, while it decreases during adulthood [21,22]. Considering different team and individual sports, the overall ratios (odds ratio (OR)) between the relatively older and younger quartiles was significant and similar in pre-adolescent (OR = 1.33) and adolescent (OR = 1.28) age groups, but disappeared in the post-adolescent stage, reasonably due to an earlier biological (i.e., stature and body mass), as well as motor skills maturation (i.e., strength, endurance, coordination, and flexibility) [3]. Moreover, the type of sport and the competition level may affect these results [3]. Considering the elite tiers of team sports in female athletes, some studies highlighted the presence of RAE at senior level in team sports, such as soccer [23], and volleyball [24] while other studies showed a symmetric distribution in birthdates [25,26,27,28]. In this regard, Sedano et al. [23] found as Spanish soccer players born in the first quartile of the year were about three times more likely to be included in the roster than players born in the last quartile of the year. Similarly, Nakata et al. [24] highlighted that Japanese volleyball players born in the first quartile were about twice more numerous than players born in the last quartile. On the other hand, the RAE was not evident in talent development and national (i.e., Swiss) soccer senior teams [27]. This trend was confirmed by Lidor et al. [25] that reported a similar number of players from each quarter in Israeli-born soccer and volleyball players, but an over-representation of players born in the third quartile in basketball. Differently, no differences in birth distribution were observed in French soccer and basketball players [26] or in Olympic basketball athletes [28].

This indicates the heterogeneity of birth distribution in the female context because of the popularity and cultural context impact on birth distribution. Interestingly, it should be noted that female sports have also shown, in some cases, atypical birth distributions where the proportion of athletes from the middle of the selection year (i.e., Q2) is higher than expected. This phenomenon, named the Q2 Conundrum [29], was observed in different studies analyzed in Smith et al.’s meta-analysis [3], where about one third of the analyzed works reported a larger magnitude of the Q2–Q4 comparison than the Q1–Q4 comparison. The cause and meaning of Q2 spike are still unclear, and this does not necessarily imply generalization in all female sport contexts. In fact, different speculations to explain this phenomenon were pointed out. The difference in birth distribution may be explained by a lower level of competition in the female than in the male sport context [3,29]. Another possible explanation is that athletes born in the Q1 compete in traditionally stereotypical female sports (i.e., tennis or swimming), leading to advantages for the Q2 athletes in non-stereotypical female sports [30], such as soccer [22]. Moreover, especially during puberty, Q1 female athletes may be competing in male leagues to seek more elite sports opportunities (i.e., stronger competition, more practice time), leading to over-representation of the Q2 in female leagues [31]. An additional possibility involves the different rules between male and female sports. For example, in female ice hockey the rules prohibit body checking [30,31]. Thus, coaches may focus their selection less on physical size and give more emphasis on sport skills that may give a different birth distribution compare to male athletes [31]. Another possible explanation of this phenomenon is that the Q2 Conundrum is present in different development stages of the various samples, suggesting that the unique environmental and sport factors may influence this trend [5]. Nevertheless, all of these considerations and ideas should warrant further consideration [31].

Therefore, even if a recent meta-analysis investigated RAE in female sport contexts, [3] only 10% of studies since 1990 focused on female-only samples [32]. This aspect highlights the need for targeted studies and models focusing on the unique issues faced by female athletes [29]. Additionally, no data are available in the Italian team sport context. Thus, the present study aimed: (i) to quantify the prevalence and magnitude of RAE in female Italian context at a professional senior level (i.e., 1st and 2nd division), focusing on basketball, soccer, and volleyball, which are the most popular team sports in Italy in terms of practitioners registered by the National Olympic Committee (CONI) (i.e., 7.1%, 23.8%, and 7.5%, respectively [33]); (ii) to verify if the RAE was present both in the early phase and/or in the later phase of the players’ career. Firstly, we hypothesized that RAE was present in all team sport at senior level. Secondly, we hypothesized that RAE was present only in the early phase subgroup of players, as we know from the literature that RAE tends to decrease as age increases.

## 2. Methods

Data in the birth dates of 1535 female athletes from the 1st and 2nd Italian division of the 2020–2021 basketball, soccer, and volleyball season were collected. Birth dates were recorded from the official database of the Italian basketball, soccer, and volleyball federations. The data collection was performed at the end of the season, after the teams’ rosters consolidation, and after possible players’ relocation among different clubs. Players’ names were removed. This study was approved by the local ethics committee of the University of Turin (Italy) and conducted in accordance with the declaration of Helsinki.

Analyses were performed considering the whole sample (1st and 2nd Italian division were merged) and the “early phase” and “later phase” players. According to a previous study [14], we considered as “early phase” those players with a lower or equal age to the respective 25° percentile of the considered sport (i.e., the first quartile of players in terms of age), while the “later phase” was identified for the rest of sample.

According to the Italian federation rules, the age-grouping cohorts are based on calendar years, for which all young players born from the 1st January to the 31st December of a calendar year are grouped together. Thus, observed birth date distributions for each quarter (i.e., Q1: January, February, and March; Q2: April, May, and June; Q3: July, August, and September; Q4: October, November, and December) and semester (S1: Q1 + Q2 and S2: Q3 + Q4) of the year were calculated. Chi-square goodness-of-fit tests were used to verify the difference between observed and expected uniform distribution (i.e., 25% for each quartile) subgroups’ quartile distributions. The magnitudes of the differences were calculated as Crammer V effect size. Threshold values for effect size statistics were V ≤ 0.17, small; V > 0.18, moderate; and V ≥ 0.29 large [34]. Additionally, comparisons between the first and last quartile (Q1 vs Q4), the second and last quartile (Q2 vs. Q4), and between the first and second semester (S1 vs. S2) were calculated using odds ratios and 95% CIs.

Subsequently, to increase the statistical power of the analysis, we decided to apply the Poisson regression for analyzing low count data [16,35]. Using this type of analysis, it is possible to investigate the RAE phenomenon more in depth, considering the birth date distribution as continuous variables [16,35]. Firstly, we calculated the week of birth (WB) of each player (e.g., in players born between 1st and 7th January WB it was 1, in players born between 8th and 14th January WB it was 2 and so on). Thus, we calculated time of birth (TB) using the following formula TB = (WB − 0.5)/52. The TB ranged between 0 and 1 and measured how far a player was born from the date of selection. Finally, Poisson regression to count data using the formula y = e ^(b0 + b1x)^ was applied to understand how the frequency of birth in a specific week (y) was explained by the TB (x). In the formula y indicated the frequency of birth in each week, x represents the TB, while b0 represented the magnifying or shrinking the scale of the entire curve and b1 the shape or degree of curvature of the (x, y) relationship [36]. The Index of Discrimination (ID), which provided the relative odds of being selected for a player born in the first versus last week of the competition year [35,36] was calculated as e^−b1^ and considered for the discussion. For all analyses, data of the first and second division were merged among the considered sports, as the tendency of RAE was similar. All data were analyzed with a custom script written in MATLAB R2021b (MATLAB, R2021b, MathWorks: Natick, MA, USA, 2021).

## 3. Results

A total of 446 (mean age = 23.4 ± 5.8 years; *n* = 224 in first division), 421 (mean age = 24.8 ± 4.9 years; *n* = 173 in first division), 668 (mean age = 24.5 ± 5.0 years; *n* = 290 in first division) elite players were analyzed in basketball, volleyball, and soccer, respectively.

The cut-off ages, according to the 25° percentile of the considered sport, for discriminating early phase and later phase subgroups were: 19 years for basketball, and 21 years for soccer and volleyball.

**Figure 1:**
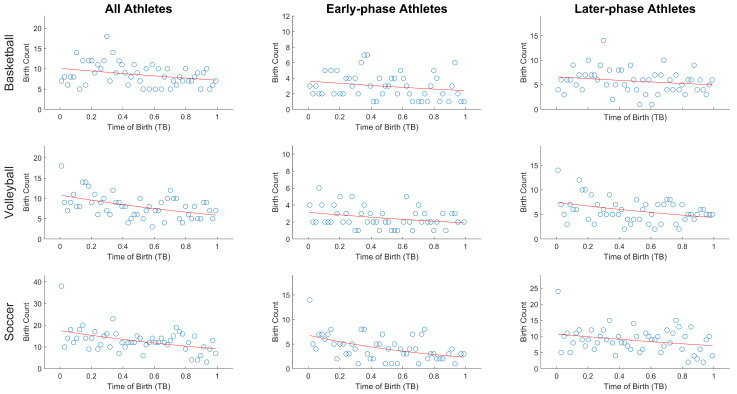
Scatter-plots of relative birth frequency by week considering all players and “early phase” and “later phase” players for basketball, volleyball, and soccer. The red line represents the best fit of the Poisson regression modeling.

Table 1 reports the relative age quartile distribution, the chi-square (χ^2^) statistics, the odds ratio, and the IDs for basketball, soccer, and volleyball considering all players, the “early phase” and “later phase” players. Figure 1 reports the scatter-plots of relative birth frequency by week considering all players and the “early phase” and “later phase” players. The red line represents the best fit of the Poisson regression modeling.

Regarding the first experimental question, i.e., when focusing on all players, both Chi-square and Poisson regression statistics showed that RAE was present in all analyzed sports with a small effect size (Crammer’s V ranged = 0.10–0.12, see Table 1). As it can be seen, analyzing the ORs presented in the Table 1 (all samples), basketball, volleyball, and soccer athletes born in the first part of the year were 1.20 to 1.62 times more represented compared to athletes born in the last part of the year. However, for basketball this ratio was statistically significant only for the S1 vs S2 ratio. In addition, the Poisson regression showed a significant effect for all considered sports (basketball: y = e ^(2.32 − 0.36x)^, R^2^ = 0.10, *p* = 0.03; volleyball: y = e ^(2.37 − 0.57x)^, R^2^ = 0.22, *p* < 0.001; soccer: y = e ^(2.87 − 0.64x)^, R^2^ = 0.21, *p* < 0.001). The ID highlighted that basketball, volleyball, and soccer athletes born right at the start of the year were 1.43, 1.78, and 1.90 times, respectively, and were more likely to be included in the rosters than those born at the end of the year.

Regarding the second experimental question, i.e., whether “early phase” players would be more prone to RAE, the results were different among sports. In soccer the Chi-square tests for “early phase” players were statistically significant (*p* = 0.001) and showed medium effect size (V = 2.69), while for the “later phase”, they were not statistically significant (*p* = 0.267). Differently, the Chi-square tests for the other sports, i.e., basketball and volley, did not show significant results (*p* > 0.056) both in the early phase and later phase subgroups.

## 4. Discussion

The present study aimed: (i) to quantify the prevalence and magnitude of RAE in the female Italian context at professional senior level (i.e., first and second division) and (ii) to discriminate in relation to the early or later phase of a career. According to our first hypothesis, RAE was found in all disciplines and the magnitude in general was small. Partially in line with our second hypothesis, in soccer RAE was evident only in early phase players and was absent in the later phase subgroup. In volleyball and basketball, the trend was less clear.

Considering the overall sample, evidence of RAE was found in each of the considered team sport with a small magnitude (Cramer’s V ranged: 0.10–0.12). The probability to be included in the rosters for an athlete born near the date of selection was 1.43, 1.78, and 1.90 times higher, for basketball, volleyball, and soccer, respectively, in comparison with the athlete born at the end of the date of selection. In other words, relative older players have more chances to be selected and reach a professional level during senior career. Even if it was not significant in term of statistical power, in basketball a spike in birth distribution in favor of players born in the second quartile was observed, suggesting a Q2 Conundrum [3,29]. However, while this phenomenon is commonly seen in less competitive disciplines, it is difficult to speculate why this occurred only in basketball.

The present results are in line with a previous study conducted for analyzing male players of Italian sport contexts, where IDs ranged from 1.48 to 2.69 [14]. Compared to male players, here, we found a lower effect size suggesting a blunted effect in females. It is possible to suggest that the lower participation in sport competition for females may reduce the competitiveness of competition and thereby moderate the magnitude of birth distribution [37]. Additionally, the sport popularity and thus the connected socio-cultural factor may enhance or limit the magnitude of the RAE. Although the lower magnitude of RAE with respect to that of male team sports, it is possible to speculate, according to selection and maturation hypothesis, that the higher muscular strength and body dimensions necessary in contact sports, such as soccer and basketball, as well as in non-contact sports, such as volleyball, may directly influence the selection process in favor of relative older female athletes. Nevertheless, it should be noted that in youth female team sports, such as volleyball, anthropometric and physical capabilities (e.g., jumping, flexibility, isometric strength, and aerobic capacity) did not vary among birth quartiles [38].

Furthermore, the present results demonstrated a different pattern compared to other national contexts, where the RAE result was negligible in basketball [26], soccer [26], and volleyball [25] but was in line with results in Spanish soccer [23] or Japanese volleyball [24]. These controversial results can be explained in relation to the countrywide, as well as to the social context, level of competitiveness, popularity, and number of active participants that affect the magnitude of RAE. In this context, soccer is the most popular sport in Italy and the data corroborate the idea that the popularity may affect the RAE [39]. Finally, socially constructed gender roles, such as stereotyped ideas of femininity, and the pressure to conform to those norms, may also pressure early maturing girls to drop out of contact sports, which would explain the lower RAE among our sample [40]. Together, these possible explanations are only speculations that should be investigated more in depth in future studies. The same is valid for the Q2 conundrum, observed only in basketball. However, the phenomenon is so nebulous that it is difficult to speculate why this occurred only in basketball.

Different fashions in birth distribution were observed when the data were re-analyzed considering players in relation to the early or later phase of their career. The most relevant results were that RAE was observed in soccer in the early phase subgroup but not in the later phase subgroup. The proportion of players born in the first and second quartile was about 2.7 and 1.9 times higher than players born in the last quartile (i.e., Q4). These results are partially in accordance with Lupo et al. [14] that reported the presence of RAE in male players during the early phase of their professional career. The only exception refers to soccer, where Lupo et al. [14] reported a presence of RAE both in the early and later career phase in male players. Conversely, our data underlined that in the later phase of a senior career the RAE disappears in all considered sports, suggesting similar probabilities to be selected for relative younger compared to relative older players.

Some limitations should be considered when interpreting these results. First, we investigated RAE only considering one competition year (i.e., season 2019–2020), thus our results may not be informative of the phenomenon in a broader view. Moreover, in our analysis we considered both Italian and foreign players playing in Italy, who probably experienced different age-category cut-offs along their junior career. Finally, the literature has indicated different RAEs according to the competitive level of the sample. Therefore, it would be interesting to split the analysis for the first and the second division. However, the sample size of the two divisions considered separately was too small to precisely detect the RAE. Therefore, even though both divisions can be considered elite-level contexts, we must acknowledge that subtle differences that may exist between them.

## 5. Conclusions

In conclusion, at a senior professional level, a small over-representation of female athletes born close to the beginning of the year is evident in all popular Italian team sports. However, this trend resulted more evidently in the first stage of a senior career, especially in soccer players, whereas it was absent in the later phase. In basketball and volleyball, data suggested a similar probability to be selected both for relative younger players and relative older players.

## Figures and Tables

**Table 1:** Relative age distribution, chi-square value, and odds ratio analysis of basketball, soccer, and volleyball players.

	Sport	Total *n*	Q1 %	Q2 %	Q3 %	Q4 %	χ2	*p*	V	ES cat.	OR Q1–Q4	OR Q2–Q4	OR S1–S2	ID (*p* Value)
All samples	Basketball	446	26.5	30.3	21.3	22.0	9.375	0.025	0.10	Small	1.20 [0.83, 1.75]	1.38 [0.83, 1.74]	1.31 [1.01, 1.71]	1.43 (*p* = 0.028)
	Volleyball	421	31.8	23.8	24.7	19.7	12.867	0.005	0.12	Small	1.61 [1.10, 2.37]	1.20 [1.09, 2.40]	1.25 [0.95, 1.64]	1.78 (*p* < 0.001)
	Soccer	668	31.0	25.1	24.7	19.2	18.719	0.001	0.12	Small	1.62 [1.19, 2.20]	1.31 [1.18, 2.21]	1.28 [1.03, 1.59]	1.90 (*p* < 0.001)
Early phase	Basketball	224	25.3	32.2	21.9	20.5	4.703	0.195	0.13	Small	1.23 [0.64, 2.39]	1.57 [0.65, 2.35]	1.35 [0.86, 2.15]	1.58 (*p* = 0.114)
	Volleyball	173	33.3	25.8	23.3	17.5	6.200	0.102	0.16	Small	1.90 [0.92, 3.96]	1.48 [0.90, 4.03]	1.45 [0.87, 2.41]	1.60 (*p* = 0.151)
	Soccer	290	36.8	25.5	24.1	13.7	22.755	0.001	0.23	Medium	2.69 [1.52, 4.76]	1.86 [1.49, 4.85]	1.65 [1.12, 2.43]	2.66 (*p* < 0.001)
Later phase	Basketball	222	27.0	29.3	21.0	22.7	5.307	0.151	0.09	Small	1.19 [0.76, 1.88]	1.29 [0.76, 1.87]	1.29 [0.94, 1.78]	1.34 (*p* = 0.143)
	Volleyball	248	30.8	23.1	25.4	20.7	7.560	0.056	0.11	Small	1.48 [0.94, 2.34]	1.11 [0.95, 2.42]	1.17 [0.85, 1.61]	1.64 (*p* = 0.014)
	Soccer	378	26.2	26.0	25.6	22.2	3.948	0.267	0.07	Small	1.18 [0.81, 1.72]	1.17 [0.90, 1.89]	1.09 [0.84, 1.42]	1.52 (*p* = 0.010)

Notes: Q1, first quartile percentage; Q2, second quartile percentage; Q3, third quartile percentage; Q4, fourth quartile percentage; χ2, Chi-square value; V, Cramer’s V effect size. OR, odds ratio and 95% confidence intervals (95% CI); Q1–Q4, first versus last quartile; S1–S2 first versus last the half year’s distribution; ID, Index of Discrimination coming from the Poisson regression analysis.

## Data Availability

The data presented in this study are available on request from the corresponding author. The data are not publicly available due to privacy reasons.
